# Separation of Plasmid DNA Topological Forms, Messenger RNA, and Lipid Nanoparticle Aggregates Using an Ultrawide Pore Size Exclusion Chromatography Column

**DOI:** 10.1021/acs.analchem.3c02944

**Published:** 2023-09-25

**Authors:** Alexandre Goyon, Shijia Tang, Szabolcs Fekete, Daniel Nguyen, Kate Hofmann, Shirley Wang, Whitney Shatz-Binder, Kiel Izabelle Fernandez, Elizabeth S. Hecht, Matthew Lauber, Kelly Zhang

**Affiliations:** † Synthetic Molecule Analytical Chemistry, Genentech, 1 DNA Way, South San Francisco, California 94080, United States; ‡ Consumables and Lab Automation, Waters Corporation, CMU-Rue Michel Servet 1, Geneva 4 1211, Switzerland; § Pharmaceutical Development, Genentech, 1 DNA Way, South San Francisco, California 94080, United States; ∥ Microchemistry, Proteomics, and Lipidomics, Genentech, 1 DNA Way, South San Francisco, California 94080, United States; ⊥ Consumables and Lab Automation, Waters Corporation, 34 Maple Street, Milford, Massachusetts 01757, United States

## Abstract

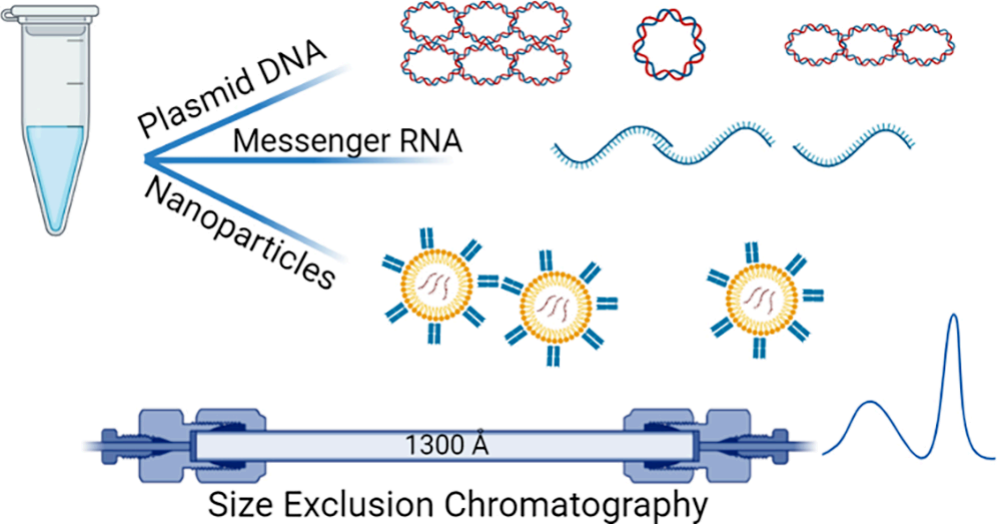

Health authorities have highlighted the need to determine oligonucleotide aggregates. However, existing technologies have limitations that have prevented the reliable analysis of size variants for large nucleic acids and lipid nanoparticles (LNPs). In this work, nucleic acid and LNP aggregation was examined using prototype, low adsorption ultrawide pore size exclusion chromatography (SEC) columns. A preliminary study was conducted to determine the column’s physicochemical properties. A large difference in aggregate content (17.8 vs 59.7 %) was found for a model messenger RNA (mRNA) produced by different manufacturers. We further investigated the nature of the aggregates via a heat treatment. Interestingly, thermal stress irreversibly decreased the amount of aggregates from 59.7 to 4.1% and increased the main peak area 3.3-fold. To the best of our knowledge, for the first time, plasmid DNA topological forms and multimers were separated by analytical SEC. The degradation trends were compared to the data obtained with an anion exchange chromatography method. Finally, unconjugated and fragment antigen-binding (Fab)-guided LNPs were analyzed and their elution times were plotted against their sizes as measured by DLS. Multi-angle light scattering (MALS) was coupled to SEC in order to gain further insights on large species eluting before the LNPs, which were later identified as self-associating LNPs. This study demonstrated the utility of ultrawide pore SEC columns in characterizing the size variants of large nucleic acid therapeutics and LNPs.

## Introduction

Plasmid DNA (pDNA) plays an essential role in cell and gene therapy and can be delivered via different methods, including electroporation for ex vivo therapy or lipid nanoparticles for in vivo delivery.^1,2^ Topological forms such as the open-circular impurities or multimers need to be characterized as they may affect the transfection efficiency.^3^ Their separation is typically performed by capillary gel electrophoresis (CGE) and anion exchange chromatography (AEC).^4^ Limitations in the AEC analysis of pDNA include adsorption issues more pronounced for the open-circular forms and limited resolution between the various topological forms.^5^ Conversely, CGE suffers from poor reproducibility and challenges associated with the identification of unknown impurity peaks.

Messenger RNAs (mRNAs) as pharmaceutical drugs have garnered significant interest since their successful use in lipid nanoparticle (LNP) vaccines to keep the coronavirus disease 2019 (COVID-19) under control and reduce the risks of life-threatening events.^6^ The accelerated approval of these vaccines by health authorities (HAs) have curbed the pandemic emergency. However, questions remain about the nature of LNP and mRNA impurities and their potential effects on efficacy and safety. Specifically, mRNA aggregates are not listed in the COVID-19 vaccine specifications and the justification of specifications is not readily available to the public.^7,8^ Both the mRNA drug substance (DS) and LNP drug product (DP) may self-associate to form aggregates. The large sizes of mRNA and LNP and extreme differences in their respective physicochemical properties are major obstacles to their analytical characterization.

The determination of LNP size and polydispersity is conventionally performed by dynamic light scattering (DLS).^7,8^ However, limitations exist with DLS, including the inability to differentiate bimodal size populations.^9^ LNP structure and morphology are commonly visualized by imaging or scattering techniques such as cryo-transmission electron microscopy (cryo-TEM), scanning electron microscopy (SEM), and small-angle X-ray scattering (SAXS).^10^ However, these techniques are not quantitative by nature, have limited throughput, or suffer from measurement challenges.^11^ There is an unmet analytical need for methods that look specifically at LNP-mRNA aggregation assemblies and potentially even the encapsulation of aggregated mRNA as cargo. It is impossible to know the impact of such alternative forms on the DP efficacy and safety without an analytical method capable of distinguishing ultrahigh molecular weight forms. It is also important that the analytical method preserves the alternative forms during analysis.

The public assessment reports by the European Medicines Agency (EMA) list the specifications of the Comirnaty (Pfizer-BioNTech) and Spikevax (Moderna) vaccines.^7,8^ Metrics include the DP size determination by DLS and purity/integrity determination by reversed-phase liquid chromatography (RP-LC) or CGE. The purity determination method by RP-HPLC typically involves a high column temperature and the use of an ion-pairing agent in combination with an organic solvent.^12^ The integrity determination of mRNA by CGE involves the use of high amounts of a denaturing agent, i.e., 4–8 M urea or formamide under nonaqueous conditions.^13,14^ In both methods, the harsh denaturing conditions used prevent detection of noncovalent aggregates.

The sizes of mRNA and LNP are usually between 100 and 500 Å^15,16^ and between 600 and 1000 Å,^17^ respectively. The large size requires the use of SEC columns with ultrawide pores, which pose heightened challenges with regard to column particle stability. The stability of a packed bed strongly depends on the average pore diameter and has to be carefully considered when large pore particles are packed.^18^ The Zenix SEC-300 column (300 Å pore size) and SRT SEC-1000 column (1000 Å pore size) are indicated for the determination of mRNA aggregates.^14^ However, aggregates of mRNA and LNPs would be unlikely to be chromatographically resolved on these columns, given that they are larger than both of these pore sizes. Some providers recently commercialized SEC columns with 1000 and 2000 Å nominal pore diameters; however, inertness of the packing material and column hardware is also important to limit the loss of nucleic acids on metal surfaces in liquid chromatography.^19^ Thus, there is an unmet need to develop new columns capable of separating these large species.

In this work, prototype ultrawide pore size SEC columns were investigated for their utility to separate ultralarge nucleic acids and their size variants. Studies were first performed in order to investigate the column’s physiochemical properties before assessing their metrics with DP applications. A heat treatment of enhanced green fluorescent protein (EGFP) mRNAs obtained from two commercial vendors was performed. The purity differences between the samples, characterized as the quantity and type of aggregates, was determined. Next, the thermal degradation of a 3.2 kilobase pairs (kbps) pDNA was evaluated using SEC and compared to an in-house AEC method. Finally, the SEC column was used to characterize a series of LNP formulations. The elution times of various LNPs were plotted against their sizes as determined by DLS. Coupling multi-angle light scattering (MALS) and differential refractometer (dRI) to SEC provided in-depth sizing and geometry information that enabled us to make insights into the order of the self-assembled species.

## Experimental Section

### Chemicals

OmniTrace Ultra grade hydrochloric acid, BioUltra grade potassium chloride, and tris(hydroxymethyl)aminomethane (Tris) base were purchased from MilliporeSigma (St. Louis, Missouri, USA). Water was generated using a Milli-Q water purification system from MilliporeSigma, and the pH of the mobile phase was determined using a SevenExcellence pH meter from Mettler Toledo (Columbus, Ohio, USA).

### Samples and Stress Conditions

#### mRNA and LNP Sample Preparation

Two EGFP mRNAs (980–996 nucleotides (nts)) were purchased from TriLink BioTechnologies (San Diego, Calfornia, USA) and GenScript (Piscataway, New Jersey, USA) at 1 mg/mL in 1 mM sodium citrate buffer (pH 6.4–6.5). The manufacturers are named “vendor A” and “vendor B” throughout the manuscript. The Cre mRNA (1350 nts) was provided by TriLink BioTechnologies at 1 mg/mL in 1 mM sodium citrate buffer (pH 6.4) and further diluted to 0.1 mg/mL with RNase-free water. The mRNAs were modified with 5-methoxyuridine (5moU) residues, 3′ poly(A) tail (100–120 nts) and 5′-cap-1. The pDNA sample was available at 1 mg/mL in water.

LNP samples were produced in-house using a NanoAssemblr Benchtop (Precision NanoSystems, BC, Canada). LNPs 1, 2, and 3 correspond to LNP samples prepared with various polyethylene glycol (PEG)–lipid compositions. Each LNP was either unconjugated (“–A”), or conjugated to Fab 1 (“–B”) or Fab 2 (“–C”).

#### mRNA and pDNA Stress Conditions

The same mRNA samples as described above were heated to study the nature of their aggregates. Samples were heated to 70 °C for 2 min.

Samples of pDNA in water were each prepared as 150 μL aliquots without dilution. Samples were heated at 50 °C and subsequently transferred to the HPLC sampler for analysis after 1, 3, 7, and 14 days.

### Instrumentation

″LC system 1″ was used for the SEC-UV analysis of the mRNAs and mRNA-LNPs. The setup was composed of a biocompatible Vanquish Binary pump module, a Split Sampler HT module, a thermostatic rapid separation (RS) column compartment, and an UltiMate 3000 diode array detector (DAD) with a 2.5 μL cell volume (Thermo Scientific, Waltham, Massachusetts). UV absorption was monitored at 260 and 220 nm (10 Hz data collection rate and 0.5 s response time).

The AEC and SEC analyses of the pDNA samples were performed using a herein described “LC system 2″. A Vanquish quaternary pump F, Split Sampler FT, and column compartment H were coupled to a DAD-FG module equipped with a 2.5 μL flow cell volume and operated at 260 nm (Thermo Fisher Scientific, Waltham, Massachusetts).

″LC system 3″ was used for SEC-MALS-dRI analysis. For separations, an Agilent 1260 series instrument with a quaternary pump, thermostated column compartment, autosampler, and DAD was employed (Agilent Technologies, Santa Clara, California). For detection, the LC-DAD system was further coupled to a DAWN MALS Detector 8 (Wyatt Technology, Santa Barbara, California) and an Optilab dRI Detector (Wyatt Technology, Santa Barbara, California) for molecular weight (MW) and radius of gyration (*R*_g_) analysis, respectively.

## Methods

### SEC

Two prototype SEC columns were manufactured by Waters Corporation (Milford, Massachusetts, USA) using 100% silica particles and a two-step silanization procedure involving the use of a hydroxy PEG (OH-PEG), trifunctional silane reagent in the first reaction, and a methoxy PEG (MeO-PEG) monofunctional silane reagent in a second, subsequent reaction step. Pore size and pore volume were measured using an AutoPore V9600 porosimeter machine from MicroMeritics (Norcross, Georgia). The average particle diameter corresponding to a 50% volume distribution was considered. The experiments were conducted at mercury pressures ranging from vacuum pressure to 400 MPa. To avoid the inclusion of the void between particles, pore size and pore volume were calculated when the intrusion pressure was greater than 2 MPa, which corresponds to a pore size around 5000 Å. To ensure accuracy, a reference sample with a known average pore diameter of 1500 Å was also measured. Particle size was measured with a Beckman Coulter's Multisizer 4e instrument (Brea, California). Surface area was measured by nitrogen adsorption using a MicroActive instrument (MicroMeritics, Norcross, Georgia), while percent carbon was measured by thermogravimetric analysis. Coverages for the OH-PEG and MeO-PEG bondings were thereby calculated according to the elemental compositions of the applied silane reagents.

The pDNA was analyzed using the column packed with “particle A” (4.6 mm I.D. × 150, 3.0 μm, with 1360 Å average pore diameter and wide pore size distribution). mRNA and mRNA-LNP analyses were performed using the column packed with “particle B” (4.6 mm I.D. x 300, 3.0 μm, with a 1275 Å average pore diameter and narrow pore size distribution). The mobile phase contained 50 mM Tris and 200 mM potassium chloride (pH adjusted to 7.5 with hydrochloric acid). The column temperature and flow rate were set to 25 °C and 0.1 mL/min. The injection volumes were set to 1.0, 2.5, and 5.0 μL for the pDNA, mRNA, and mRNA-LNP samples, respectively. For MALS-dRI analyses, LC system 3 was used with a 4.6 mm I.D. × 300 mm column packed with “particle B”. Injection volumes were set to 20–30 μL for mRNA-LNP samples, and all other method conditions were identical with those in the SEC-UV experiment.

### AEC

A BioPro QF column (4.6 mm I.D. × 100 mm, 5.0 μm, nonporous) was purchased from YMC America (Fort Devens, Massachusetts, USA). Mobile phase A contained 50 mM Tris in water (pH 7.5), while mobile phase B contained 50 mM Tris and 1.0 M potassium chloride in water (pH 7.5). Column temperature and flow rate were set to 20 °C and 0.7 mL/min. The autosampler was operated at 5 °C, and the injection volume was set to 5.0 μL. The mobile phase B composition was increased from 75 to 100% over 20 min and maintained at 100% for 2.5 min, followed by re-equilibration of the column for 7.5 min at 75% mobile phase B.

### Software

LC systems 1, 2, and 3 were controlled by Chromeleon 7.2.10 (Thermo Fisher Scientific), Empower 3 Software build 7.50.00 (Waters Corporation), and ChemStation A.01.04 (Agilent Technologies), respectively. Astra 8 Software was used for data analysis in SEC-MALS-dRI. The dRI detector was used for MW determination with the refractive index increment (d*n*/d*c*) value of 0.165 mL/g.

## Results and Discussion

### Physicochemical Characterization of the Prototype Ultrawide Pore SEC Columns

**Figure 1:**
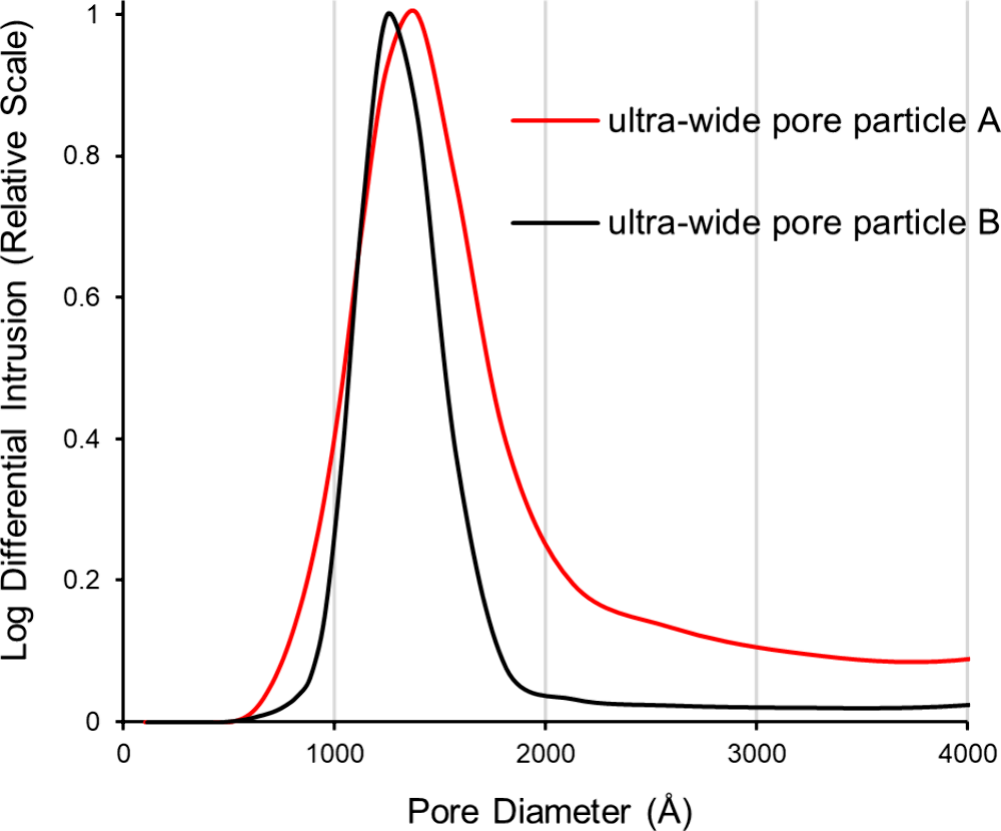
Experimentally measured pore diameter distributions of the prototype ultrawide pore SEC materials.

The average pore diameters were measured as 1360 and 1275 Å for ”particle A” and “particle B”, respectively. The ”particle A” material possesses a broad pore size distribution, while ”particle B” has a narrow pore distribution (Figure 1). Table 1 lists the physicochemical properties of the two types of particles. Both were treated with a two-step PEGylation silane reaction.

**Table 1:** Physicochemical Properties of the Two Prototype Ultrawide Pore SEC Particles

	”particle A”	”particle B”
average pore size (Å)	1360	1275
pore volume (mL/g)	0.61	0.66
average particle diameter (μm)	2.49	2.47
surface area (m^2^/g)	12.4	16.0
carbon content after OH-PEG bonding (wt %)	0.46	0.56
carbon content after MeO-PEG bonding (wt %)	0.65	1.12
OH-PEG coverage (μmol/m^2^)	1.36	1.28
MeO-PEG coverage (μmol/m^2^)	0.68	1.68

The two types of particles were then packed into low adsorption column hardware. The surface of the hardware is composed of an ethylene-bridged siloxane polymer that is formed on metal substrates using a vapor deposition process.^19^ Besides a reduction in electrostatic interactions versus metal surfaces (i.e., stainless steel hardware), the ethylene-bridged hybrid surface is predicted to exhibit lower hydrophobicity than some alternative polymeric surfaces. Therefore, we expected that such column hardware would be well suited to the analysis of challenging nucleic acids.

### Characterization of Multiple Nucleic Acid Products and Attributes

mRNA and pDNA are characterized by their long length, > 1000 nts for mRNA and >3 kbps for pDNA, and hydrophilic properties. However, they differ by the type of impurities commonly observed. mRNAs are single-stranded RNA molecules that can self-associate to form secondary structures such as hairpins and loops and interact with other mRNA molecules resulting in the formation of aggregates. The main impurities reported in the literature thus far are shortmer impurities missing the polyA tail for mRNA,^20^ while topological forms and multimers are often observed with pDNA products.^5^ Topological forms have the same MW with the desired supercoiled pDNA product but differ in the arrangement of their strands, such as open-circular impurities. The large size of both mRNA and pDNA and their extreme polarity and charge have been an obstacle to their characterization by SEC. Their large size requires ultrawide pore columns, while the presence of numerous phosphate groups requires column materials that limit nonspecific interactions.^19^

#### Determination of mRNA Aggregates

**Figure 2:**
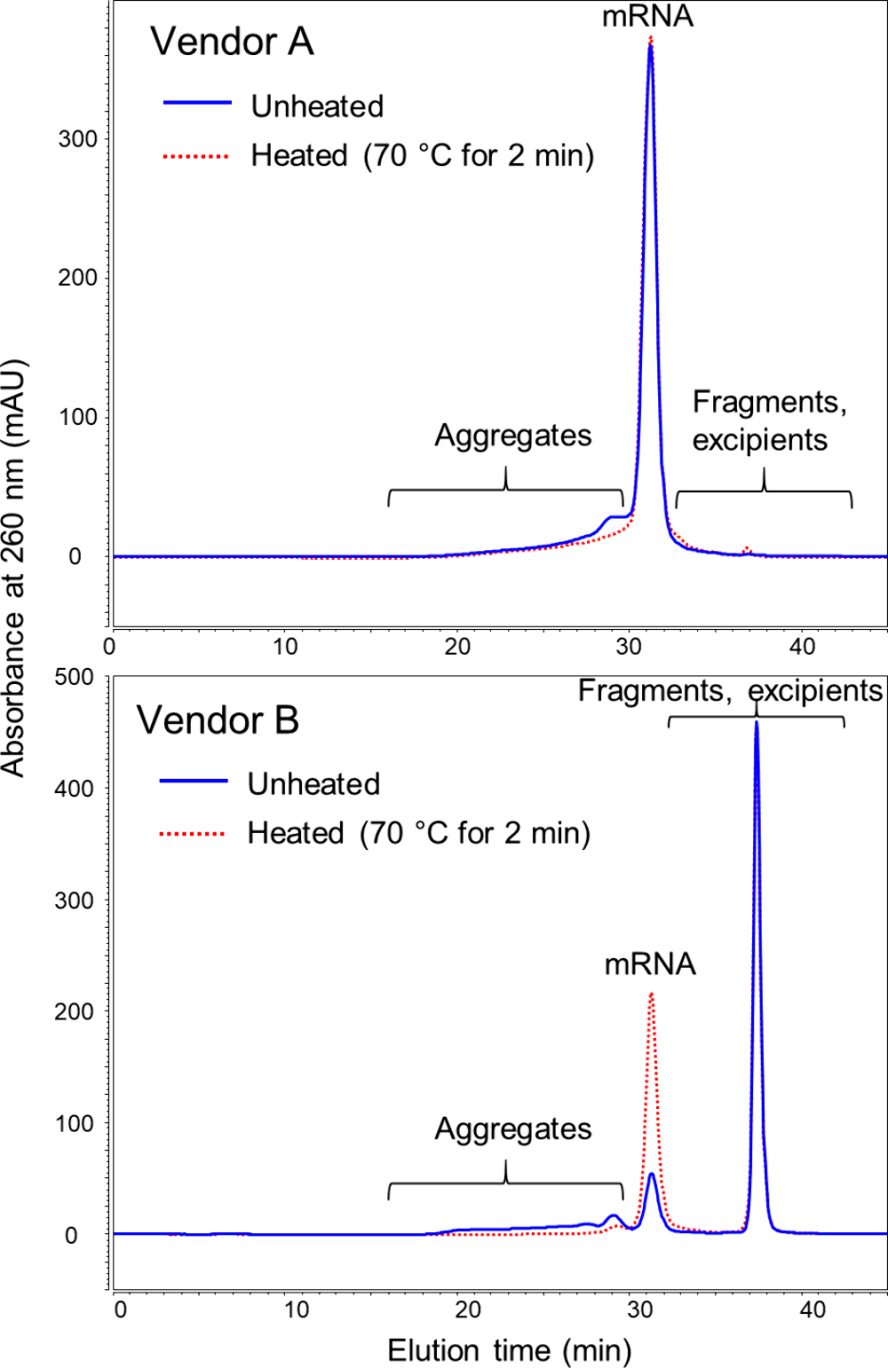
SEC-UV profiles obtained for EGFP mRNA supplied by two vendors. The profiles were obtained before heating the samples (blue traces) and after heating (red traces). The mRNAs have the same nominal concentration of 1 mg/mL and similar length (980–996 nts).

The presence of aggregates was compared for EGFP mRNA produced by different manufacturers (Figure 2).

Significant differences were observed between the unstressed samples from two different vendors (blue traces in Figure 2). The material supplied by vendor B had a significantly reduced peak area compared to vendor A for the mRNA peak eluting at 31.26 min. In addition, vendor B’s sample showed a greater amount of aggregates (59.7 vs 17.8%), with species eluting between 17.25 and 30.20 min. A major peak eluted at 37.39 min, corresponding to smaller nucleotide fragments or excipients.

The nature of the aggregates was also investigated by performing a heat treatment (red traces in Figure 2). With the vendor A material, the intact mRNA absolute peak area remained similar upon heating (2% relative difference) while the mRNA peak area increased 3.3-fold for the mRNA supplied by vendor B. Heating vendor A’s mRNA resulted in the formation of an additional peak at 36.80 min (0.8% relative peak area), suggesting the formation of fragments. Identification of these species was not pursued, although these peak fractions might be amenable to analysis by denaturing IPRP or CGE. A significant reduction of aggregates from 59.7 to 4.1% was observed for the mRNA supplied by vendor B after the heat treatment, while a more modest reduction of aggregates was observed with the mRNA supplied by vendor A (17.8 to 15.1%, respectively). These results suggest that the aggregates detected in the vendor B’s mRNA were predominantly noncovalent in nature. Given that the heated samples were prepared 24 h before being analyzed by SEC-UV, we hypothesize that the dissociation of the mRNA aggregates may be irreversible or may reform at a very slow kinetic rate. However, further studies would be needed to confirm the hypothesis.

The differences in mRNA and aggregate content between the two mRNA samples highlight the value of SEC to compare the quality of materials at a batch and vendor-specific levels. SEC facilitated characterization of the nature of the aggregates, which differed in elution time and size. The heat treatment experiment showed the importance of using a nondenaturing method to quantify the amount of aggregates. It is unclear at this time how the heat treatment may affect the mRNA structures and whether it would affect the potency of the final product. The fast sample manipulation could also ensure mRNA content consistency between different batches as well as reduce potential safety risks that may be associated with the aggregates, i.e., immunogenicity and off-target toxicity.

#### Determination of pDNA Topological Forms and Multimers

**Figure 3:**
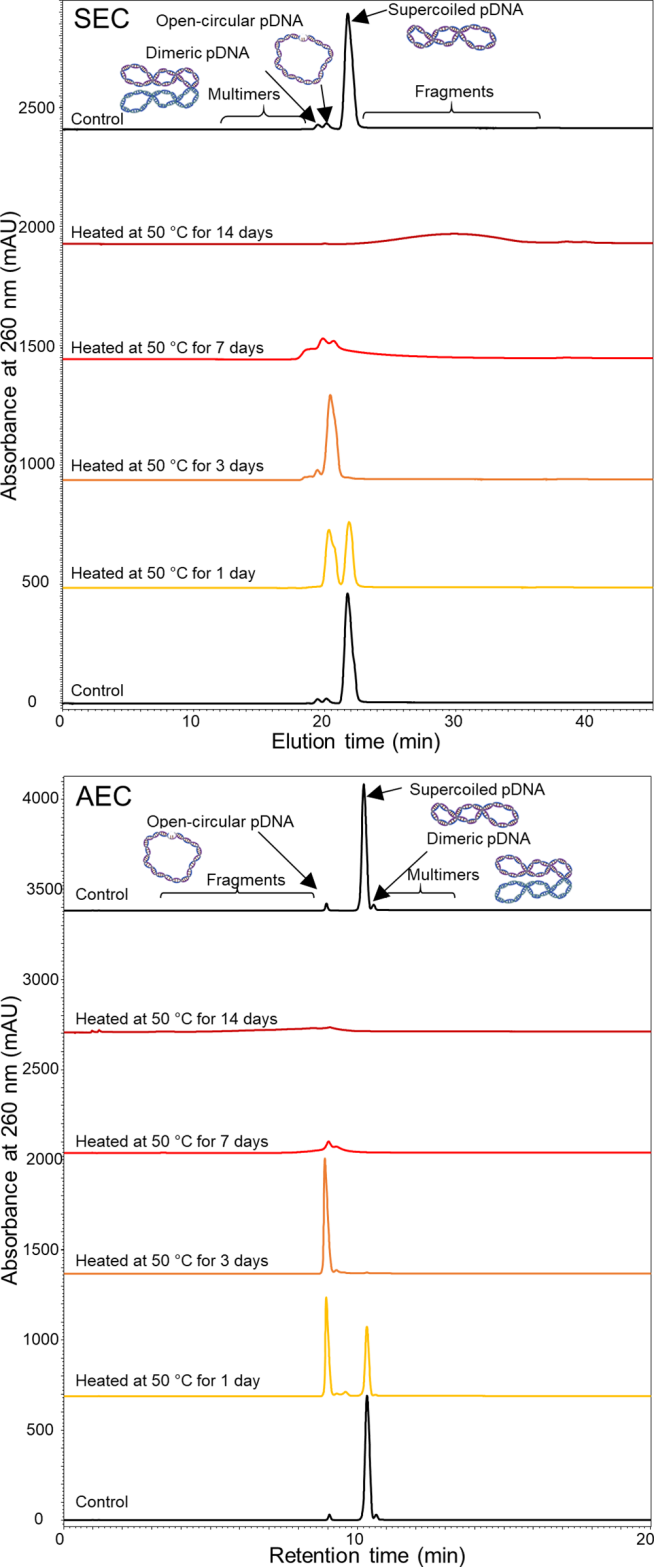
SEC and AEC profiles of a pDNA sample stressed at 50 °C for up to 14 days.

The analytical characterization of plasmid impurities can be used to predict the transfection efficiency and ultimately guide the control strategy. Plasmid DNA topological forms and multimers are commonly separated by agarose gel electrophoresis (AGE), CGE, and AEC. The supercoiled form often referred to as covalently closed circular (ccc) form is preferred while other forms are considered unusable forms of pDNA.^4^ Sousa et al. demonstrated that the transfection efficiency was improved by using supercoiled pDNA.^21^ However, arguments exist against the necessity of having a high supercoiled content depending on plasmid use.^22^ The USP 35 <1047> Gene Therapy Products guidance released in 2012 lists AEC for the quantification of the individual topological isoforms.^4^ However, adsorption issues have been observed and are a disproportionate problem in particular for the open-circular forms, which were underestimated in several studies.^5,23^ The development of ultrawide pore SEC columns would enable the characterization of these undesired nucleic acid forms. Figure 3 shows the SEC and AEC profiles of a pDNA and the changes in its profile after exposure to thermal stress.

Two major impurity peaks were observed for the unstressed pDNA samples on both the SEC and AEC profiles (black traces in Figure 3). Broader peaks were observed on the SEC profiles due to the nonretentive nature of the size-based separation and the poor diffusion of the large pDNA. SEC and AEC separations achieved some degree of orthogonality. In the SEC profile, the two impurity peaks eluted before the main peak (*t*_E_ = 21.71 min). In the AEC trace, the main peak eluted at 10.32 min, with one impurity peak eluting after and one eluting before. The relative peak areas of the two impurities were 2.3% (*t*_E_ 19.44 min), 2.9% (*t*_E_ 20.12 min) in SEC and 2.5% (*t*_R_ 9.05 min), 2.7% (*t*_R_ 10.65) in AEC. The SEC data indicated that both impurities had larger hydrodynamic radii than the supercoiled pDNA, and the AEC data indicated that one impurity had an increased local net charge distribution and one had a decreased local net charge distribution relative to the original structure. Multimers of DNA can bear a greater number of charges, suggesting that this corresponded to the AEC peak at 10.65 min. The degradation trends were evaluated in order to gain further insights into the formation and structures of these impurities.

After 1 day (yellow traces on Figure 3), a significant increase of the first eluting impurity by AEC (*t*_R_ 9.05 min) and second eluting peak by SEC (*t*_E_ 20.12 min) was observed. The open-circular impurity forms during thermal stress of pDNA.^23^ The formation of the open-circular form at elevated temperature has been attributed to the presence of residual endogenous nucleases.^24^ Thus, the second and growing AEC impurity peak at 9.05 min was assigned to the open-circular impurity. This correlated well with the SEC data. The open-circular form on the SEC trace grew to a 52% relative peak area and a 51% relative peak area, as determined by AEC. The open-circular impurity was the most abundant species observed on the SEC profiles after 3 days (orange traces on Figure 3).

**Figure 4:**
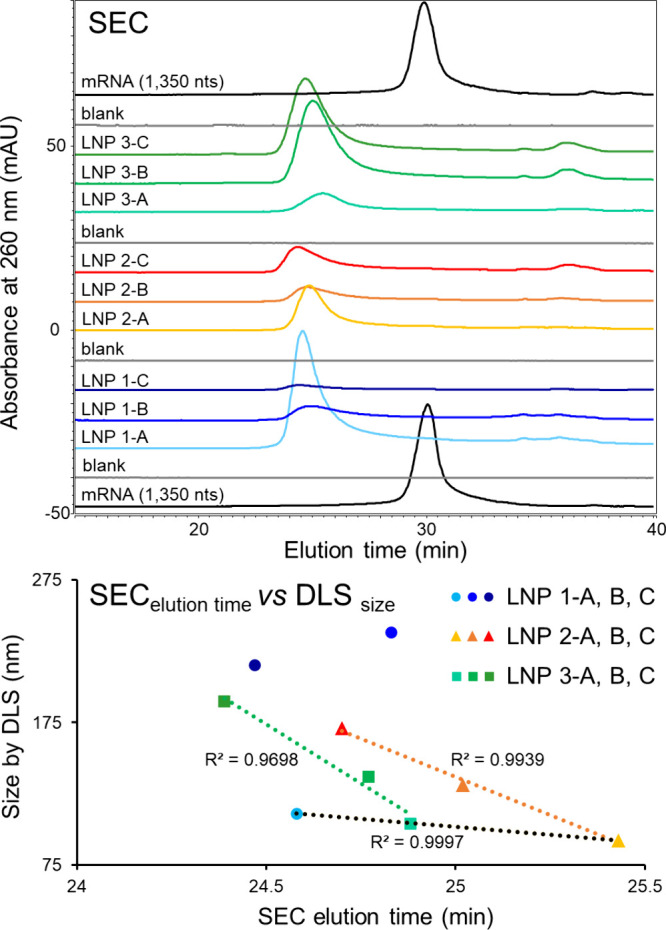
SEC-UV profiles of mRNA-LNP samples and the corresponding Cre mRNA standard. The LNP sizes determined by DLS were plotted against the SEC elution time of the main peak. LNPs 1, 2, and 3 correspond to LNPs prepared with various PEG-lipid compositions. Each LNP was either unconjugated (“-A”), or conjugated to Fab 1 (“-B”) or Fab 2 (“-C”).

A significant decrease in the amount of DNA species was observed after 7 days (red traces in Figure 3), which could be due to the formation and precipitation of insoluble species. A large amount of impurities were also observed to elute earlier than the open-circular species on the SEC profiles, which could suggest the formation of multimers. The formation of a secondary degradation of impurities was observed after 14 days (dark red traces on Figure 3) with the presence of a late eluting peak on the SEC profile (*t*_E_ > 21 min) and early eluting peak on the AEC profile (*t*_R_ < 10 min). The broad peak observed on both AEC and SEC profiles was associated with heterogeneous fragment size populations. The production of fragments after the appearance of denaturation products would be expected later in the thermal stress period. Despite the possible formation of heterogeneous impurities, the AEC and SEC profiles of the control sample (black traces in Figure 4) bracketing the stressed sample injections remained the same. This demonstrated the suitability of the methods and instrumentation.

Sample carryover was evaluated for the open-circular form, supercoiled form, and dimeric impurity. Using AEC, the relative peak areas of the open-circular form, supercoiled form, and dimeric impurity in a blank sample were 3.9, 0.2, and 4.3% to those integrated for the corresponding peaks of a pDNA standard injected before. With SEC, the relative peak areas were reduced to 1.2, 0.5, and <0.1% for the dimeric impurity, open-circular form, and supercoiled form, respectively. The low carryover achieved by SEC could help in improving the accuracy of open-circular impurity measurements.

Overall, the SEC and AEC traces provided orthogonal information that enabled more certain identification of the impurities present in the pDNA samples. The employed prototype ultrawide pore SEC columns showed powerful resolution of multimeric species that would likely not be achievable on columns packed with smaller pore size particles.

### Determination of Multiple Attributes for mRNA-LNPs

Multiple attribute methods that facilitate simultaneous measurements can streamline analytical characterization. The determination of free mRNA DS present in the final mRNA-LNP DP informs the efficiency of the encapsulation process and stability of the DP. As discussed in the introduction, the large size of both the mRNA and LNP is an obstacle to their separation using SEC columns with a pore size lower than 300 Å. Figure 4 shows the SEC profiles obtained for a Cre mRNA standard (1350 nts) and various mRNA-LNP samples. The various LNPs represent real-world samples and are more heterogeneous and complex in nature than the monodisperse markers usually tested to characterize SEC materials.

The Cre mRNA standard eluted at 30.08 min (black traces on Figure 4) while the main peak of the various LNPs eluted between 24.39 and 25.43 min (blue, orange, and green traces on Figure 4), demonstrating the ability of the SEC columns to resolve the free mRNA from the intact LNPs. Interestingly, various elution times were observed for the LNP 1, 2, and 3 batch traces. The largest differences in elution times were observed between LNPs with or without conjugation to Fab ligands (LNPs B–C), which eluted earlier than the unconjugated LNPs (LNPs A).

We then further evaluated whether these elution time differences could be correlated to the LNP size, as determined by DLS (Figure 4B). First, the elution times of the unconjugated LNP1-A, LNP2-A, and LNP3-A correlated with their size determined by DLS (*R*^2^ > 0.99), demonstrating the effect of changing the PEG-lipid on the LNP size. In addition, for the LNP2 and LNP3 series, SEC elution times were found to be correlated with the size measured by DLS (*R*^2^ > 0.96). However, there was no correlation with LNP 1 series. This may be related to the difference in PEG-lipids used to synthesize LNPs 1, 2, and 3. PEGylation of lipids helps to prevent or limit the aggregation of LNPs by shielding their hydrophobic surface.^25^ It was particularly interesting to see that the size determined by DLS for LNP 1-B and LNP 1-C was greater than any other LNP, and a multimodal size distribution was observed for the LNP 1-B in DLS. It might be that sample heterogeneity affected the DLS measurements,^9^ thus causing the poor correlation for LNP 1 series in Figure 4.

**Figure 5:**
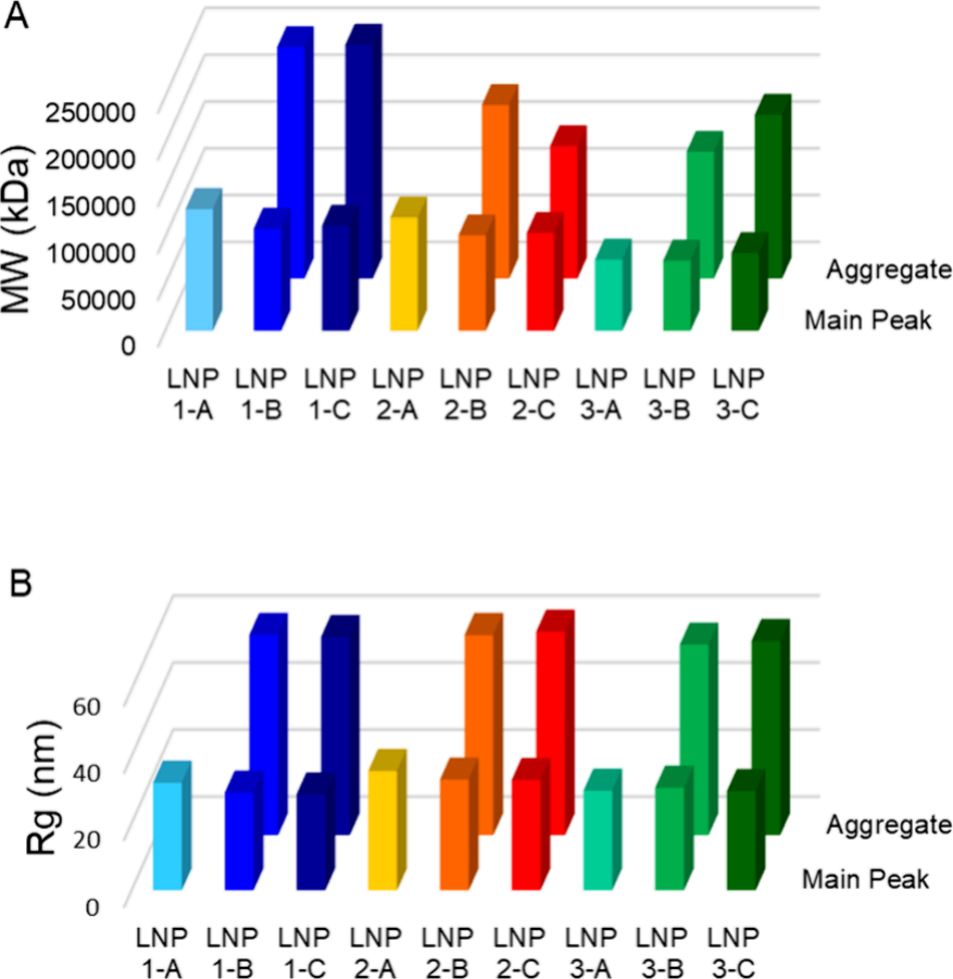
SEC-MALS-dRI results of mRNA-LNP samples (LNP As) and the corresponding Fab-conjugated mRNA-LNP samples (LNP Bs and Cs). (A) MW and (B) *R*_g_ analyses by SEC-MALS-dRI.

We also observed a minor prepeak in the SEC chromatograms of all LNPs after surface Fab conjugation (LNPs B–C). Combining the MALS detector and a concentration detector, dRI, can measure the light scattered by an analyte in multiple angles and determine their MW and shape. Therefore, SEC-MALS-dRI investigations were performed in order to gain additional insights from the MW and the size analysis of the LNPs (Figure 5). The MW analysis suggested that the prepeak had a ∼1-fold increase in MW compared with the main peak (Figure 5A). In addition, the radius of gyration (*R*_g_), the center of mass, also doubled after conjugation (Figure 5B). Given that the hydrodynamic radii of Fab ligands are normally smaller than 50 Å,^26^ coating a layer of Fab ligands onto a ∼1000 Å LNP would be unlikely to cause the size to increase significantly. Therefore, the *R*_g_ increase was attributed to particle–particle aggregation. Although the unfunctionalized mRNA-LNPs (LNP As) remained as monomeric particles due to the incorporation of PEGylated lipids, the Fab functionalized mRNA-LNPs (LNP B–Cs) showed a higher tendency to aggregate. The presence of Fab ligands on the particle surface modified the LNP surface charges and increased surface hydrophobicity, thus causing more interparticle association. This study demonstrates the versatility of SEC analysis in combination with alternative detection techniques, providing online MW/sizing analysis and thus impurity (i.e., aggregates) identification for mRNA-LNPs. The SEC method can capture a small amount of particle aggregate that may not be resolved by alternative sizing techniques such as DLS. Detection of particle aggregates in various formulations can support process development to optimize conjugation chemistries and improve particle physical stability.

## Conclusions

To our knowledge, this study is the first to characterize mRNA and LNP aggregates and plasmid topological forms and multimers by SEC. Prototype SEC columns packed with sub-5 μm particles and ultrawide pore morphologies were utilized to achieve these results. The physicochemical properties of the SEC packing materials were systematically evaluated. It was found that their average pore size and pore size distribution are well suited to the analysis of mRNA, LNP aggregates, and plasmid topological forms and multimers.

Significant differences in aggregate content (59.7 vs 17.8%) were observed for EGFP mRNA supplied by different manufacturers. Interestingly, most aggregates from vendor B were dissociated or may reform at a very slow kinetic rate upon heat treatment. On the same time scale, the main mRNA peak area increased 3.3-fold. The degradation trends of a pDNA (3.2 kbps) were compared using SEC and AEC. The information provided by orthogonal chromatographic modes enabled the identification of open-circular and multimer impurities that would have remained ambiguous when characterized by a singular method. The comparison of AEC and SEC profiles revealed a multistep degradation process involving the formation of (1) open-circular forms, (2) large multimers and insoluble species, and (3) fragments. Lower sample carryover was observed using SEC in comparison to AEC.

Multiple critical attributes of complex mRNA-LNP samples conjugated to Fab ligands were investigated using the ultrawide pore SEC columns. The mRNA was separated from the intact LNPs. Correlations between SEC elution time and size measured by DLS were established for two LNP series (*R*^2^ > 0.96). Further identification of a prepeak eluting before the main peak of LNPs conjugated to Fab ligands was performed using SEC-MALS-dRI, suggesting the presence of particle aggregates.
